# Oncogenic viruses associated with vulva cancer in HIV-1 patients in Botswana

**DOI:** 10.1186/1750-9378-9-28

**Published:** 2014-08-24

**Authors:** Kenneth O Simbiri, Hem C Jha, Mukendi K Kayembe, Carrie Kovarik, Erle S Robertson

**Affiliations:** 1 Department of Microbiology and Immunology, Upstate Medical University, R2120A, Weiskotten Hall, 750 East Adams Street, Syracuse, NY 13210, USA; 2 Department of Microbiology, Abramson Cancer Center, Tumor Virology Program, Perelman School of Medicine at the University of Pennsylvania, 202A Johnson Pavilion, 3610 Hamilton Walk, Philadelphia, PA 19104-6076, USA; 3 National Health Laboratory, Gaborone, Botswana; 4 Botswana-University of Pennsylvania Partnership, Gaborone, Botswana; 5 Department of Dermatology, University of Pennsylvania Perelman School of Medicine, Philadelphia, PA, USA

**Keywords:** HIV-1, EBV, HPV, KSHV, OSSN

## Abstract

**Background:**

Oncoviruses such as HPV, KSHV, and EBV have been reported in patients with HIV infection and AIDS. How oncovirus-associated cancers rise in AIDS patients has not been fully established. The purpose of our study was to identify the viral agents in vulvar cancer and to assess their contribution to pathogenesis.

**Method:**

We retrospectively identified a total of 13 vulva tissue samples from HIV-1 positive and 9 vulvar samples from HIV-1 negative patients from the Botswana National Health Laboratory in Gaborone, Botswana, a Southern African country with a high incidence of HIV. We utilized PCR and IHC to identify HPV, EBV, KSHV, and JC virus in FFPE preserved tissue samples.

**Results:**

Using the GP5^+^/GP6^+^ primer set we detected several HPV types in tissue samples. EBV was detected in all of the positive cases (100%) and in most of the negative cases (89%). KSHV was detected in 39% of the HIV-1 positive samples and in 11% of the negative samples, and no JC virus was detected in any of the samples.

Using IHC we demonstrated that LANA was expressed in 61% of the positive samples and in 44% of the negative samples. The ubiquitous EBV was more consistently expressed in negative cases (100%) than in positive cases (69%). Interestingly, the HPV-16 E6 transcript was detected in 56% of the negative samples compared to 31% of the positive samples. However, the cell cycle protein P21 used as a surrogate marker for HPV was detected in 77% of the positive samples and in 44% of the negative samples, while VEGF signals were similar in both positive (92%) and negative samples (89%).

**Conclusion:**

Our study, suggests that in Botswana, vulvar squamous cell carcinoma (VSCC) is associated with oncogenic viruses present in the niche but the contribution and progression may be regulated by HPV and other immunosuppressive infections that include HIV-1.

## Introduction

There are two types of vulvar cancer. The first type that develops from intraepithelial neoplasia caused by human papillomavirus (HPV) infection, shows multifocality, and is increasing in prevalence among young women and especially those with multiple co-infections including HIV [1]. The second type, differentiated or simplex, is more often seen in older women with p53 alterations and likely develops from non-neoplastic epithelial disorders as a result of chronic inflammation [1]. Basta et al., observed strong correlation between patients younger than 45 years and HPV, cigarette smoking, having more than two sexual partners, sexual initiation before age 19 years, and low socioeconomic status [2]. In patients older than 45 years, there was correlation between vulvar intraepithelial neoplasia (VIN) and non-neoplastic epithelial disorders (VNED), residence in rural area, low economic status, menopause before age 45, poor hygiene, endocrine disorders, and low serum vitamin A levels. Pilotti et al. in a study of patients with intraepithelial lesions reported a strong correlation with the presence of HPV-16 DNA, cigarette smoking, and herpes simplex cofactor [3]. Further they reported that alteration of p53 by either interaction with viral oncoproteins or somatic mutations could be crucial to the pathogenesis of vulva carcinomas, and that p53 mutations are mainly associated with disease progression [4]. In another study looking at HPV infection and co-infection with HSV-2 and *Chlamydia trachomatis* in vulvar intraepithelial neoplasia Kwasniewska et al. reported that 73% were HPV-16 positive, 14.63% were HSV-2 positive, and 14.63 *Chlamydia trachomatis* positive [5]. Hampl et al., in a German study of 224 patients with vulva cancer identified between 1/1980 and 6/2007 noted a significant shift in age with the initial period having 11% of the women being under 50 years while the third period had 41% of the women being less than 50 years, and that two thirds of the women under 50 years were HPV positive [6]. They also noted that the tumor localization had changed from labia to the area between the clitoris and urethra [6].

In sub-Saharan Africa there has been a paucity of vulvar carcinoma studies and in the referenced studies the rates of vulvar carcinomas are low and recently more associated with HIV infection [7-9]. Importantly, these studies have not investigated the link of vulvar cancer and viral infections [10-12].

The aim of this study was to identify the oncogenic viruses in vulvar carcinoma tissues in HIV positive and negative vulvar cancer patients from Botswana and to assess the differential expression of cell cycle proteins and growth factors that may be associated with transformation of cells. Further, from strong evidence associating these proteins with G1 phase arrest dysregulation and angiogenesis in some cancers we wanted to confirm their expression in VSCC [13-15].

## Materials and methods

We retrospectively identified a total of 13 vulva tissue samples from HIV-1 positive and 9 vulvar samples from HIV-1 negative patients from the Botswana National Laboratory in Gaborone, Botswana (Table 1). Botswana Ministry of Health, Princess Marina Hospital, and University of Pennsylvania IRB approved this study (protocol # 812041). Princess Marina Hospital resident pathologist and University of Pennsylvania dermatologist histologically confirmed all tissue samples as vulvar squamous cell carcinoma (VSCC). Tissue samples were procured from women above age eighteen with in situ or invasive squamous cell vulvar cancer. Tissue samples were prepared from the paraffin embedded samples by cutting 5 μm thick sections using different blades to limit cross contamination, followed by de-paraffinization and extraction of the DNA. Total cellular DNA was extracted from the vulvar tissues by digestion with proteinase K and phenol-chloroform extraction as previously described [16]. PCR analysis was performed as previously described [17-21] with each virus specific primer including HPV types using consensus primer GP5+/GP6+ that detects more than 20 HPV types including, 11, 16, 18, 31, 33 and 45; EBV, KSHV, and JC virus. The PCR assay was performed with primers HPV-L1 (150 bp); 5’TTTGTTACTGTGGTAGATACTAC-3’ , 3’-CTTATACTAAATGTCAAATAAAAAG-5’; EBV-BamH1W (129 bp); 5’- CCAGACAGCAGCCAATTGTC-3’ , 3’-GGTAGAAGACCCCCTCTTAC-5’; KSHV-ORF-73 (293 bp); 5’-CCATCTCTTGCATTGCCAC-3’, 5’-AACTACGGTTGGCGAAGTCA-3’; and JC VP2/VP3 (133 bp); 5’GAAGAACCCAAAACTATTTGTTGAAA-3’, 5’-GCCTAACTGGAGACAATCTAGAATAATAGTC-3’. The PCR conditions were as follows: For HPV- GpP5^+^/GP6^+^ 94°C for 5 minutes, 94°C for 30 seconds, 48°C for 30 seconds, and 72°C for 30 seconds, for 40 cycles; elongation at 72°C for 5 minutes; and then incubation at 4°C. For EBV-94°C for 5 minutes, 94°C for 30 seconds, 47°C for 30 seconds, and 72°C for 30 seconds, for 40 cycles; elongation at 72°C for 5 minutes; and then incubation at 4°C. For KSHV- 94°C for 5 minutes, 94°C for 30 seconds, 52°C for 30 seconds, and 72°C for 30 seconds, for 40 cycles; elongation at 72°C for 5 minutes; and then incubation at 4°C. The PCR products were run on 2.5% agarose gel at 100 V for 1 hour.

**Table 1:** Patient characteristics

**HIV negative**						
	**Age**	**Bx site**	**Diagnosis**	**HIV status**	**HAART Dx**	**CD4 counts**
1	32	Vulva	SCC	-	NO	NO
2	79	Vulva	SCC	-	NO	NO
3	35	Vulva	SCC	-	NO	NO
4	52	Vulva	SCC	-	NO	NO
5	79	Vulva	SCC	-	NO	NO
6	69	Vulva	SCC	-	NO	NO
7	62	Vulva	SCC	-	NO	NO
8	42	Vulva	SCC	-	NO	NO
9	70	Vulva	SCC	-	NO	NO
**HIV positive**						
1	39	Vulva	SCC	+	NO	676
2	37	Vulva	SCC	+	NO	477
3	41	Vulva	SCC	+	NO	347
4	49	Vulva	SCC	+	NO	166
5	35	Vulva	SCC	+	NO	157
6	32	Vulva	SCC	+	NO	112
7	35	Vulva	SCC	+	NO	350
8	41	Vulva	SCC	+	YES	497
9	35	Vulva	SCC	+	YES	303
10	40	Vulva	SCC	+	NO	854
11	29	Vulva	SCC	+	YES	189
12	48	Vulva	SCC	+	YES	751
13	32	Vulva	SCC	+	YES	254

The table shows age, patient diagnosis, the retroviral therapy status, and CD4 counts of each patient in the study. All patients were diagnosed with squamous cell carcinoma of the Vulva.

+= HIV positive; NO=HAART not administered and CD4 count not done.

In order to prevent PCR contamination we prepared PCR reagents before each assay in a master mixture that was then aliquoted. We prepared the master mixture, extracted the DNA and added the template to the PCR mixture in one area. The thermal cycling was performed in a separate area. We took precautions against contamination by using aerosol-barrier-protected pipette tips, frequent changes of gloves, and repeated decontamination of surfaces with sodium hypochlorite.

Our sample extracts including positive controls and a water specimen as an additional negative control to rule out contamination of the reagents by aerosols, were also subjected to PCR as described above. To support the PCR findings for the presence of the oncogenic viruses, HPV, EBV, and KSHV while concomitantly monitoring changes associated with oncogenesis through expression of known surrogate markers in tissues we performed immunohistochemistry on 5 μm thick paraffin- embedded sections to detect virus specific antigens, cell cycle proteins and vascular endothelial growth factor (VEGF) [9]. We used commercial antibodies to HPV 16-E6 and E7 and HPV 18-E6 and E7 (DAKO Inc., Carpentaria, CA), monoclonal antibody S12 for detection of EBV-LMP1, monoclonal antibody derived from KSHV encoded LANA [22], Cyclin D antibody (Santa Cruz, TX), p53 antibody (Santa Cruz, TX), VEGF antibody (Abcam, MA), and p21 antibody (Abcam, MA).

### Data analysis

The data for this study was collected in Excel and imported into a statistical software program. Analysis encompassed determination of proportions (MedCalc Software bvba MedCalc Software, Ostend, Belgium) was used for analysis.

## Results

### Study population characteristics

In this study tissue blocks from 9 HIV negative patients and 13 HIV positive patients were used (Table 1). We performed PCR using different primers of HPV types, EBV, KSHV, and JC virus. A larger percentage of high-risk HPV types were detected in the tissues than low risk HPV types. Using the GP5^+^/GP6^+^ primers we detected HPV in 67% of the negative cases and 54% of the positive cases (OR = 0.57, CI = 0.32-1.02, P = 0.06) (Table 2). HPV-18 was not detected in negative cases and in only 1 (8%) positive case. Though HPV-18 is usually detected in fewer cases than HPV-16, the low number of positive cases was surprising in this unique population. Equally surprising was the detection of HPV-16 at similar levels in the negative cases and positive cases 78% and 77% respectively (OR = 0.94, CI = 0.48-1.83, P = 0.86) indicating the ubiquity of HPV-16 in this population (Table 2). More HPV-45 (56%) was detected in negative samples than positive samples (39%) (OR = 0.50, CI = 0.28- 0.88, P = 0.01) while HPV-33 was undetectable in negative samples and in only one positive case (OR = 18.47, CI = 1.05-324, P = 0.04). Moreover HPV-31 was detected to a greater degree in positive samples (85%) than negative samples (67%) (OR =1.94, CI = 0.94-4.01, P =0.07). The low risk HPV-11 was detected in fewer negative samples than positive samples (OR = 2.41, CI = 1.10-5.27, P = 0.02) while HPV-6 was the same in the negative samples (44%) and positive samples (46%) (OR = 1.08, CI = 0.62-1.89, P = 0.78). EBV was detected in all the positive cases and in 89% of the negative cases (OR = 25.82, CI = 1.50-444.6, P = 0.02). KSHV was detected in 39% of the positive cases and 11% of the negative cases (OR = 5.17, CI = 2.45-10.8, P < 0.01), and no JC virus was detected in any patient sample (Table 2).

**Table 2:** PCR summary of HIV-1 negative and positive vulva cancer samples

**Negative**	**GP5+/6+**	**HPV-18**	**HPV-16**	**HPV-45**	**HPV-33**	**HPV-31**	**HPV-11**	**HPV-6**	**EBV**	**KSHV P1/P2**	**KSHV F/R**	**JC**
1	+	-	-	-	-	-	-	-	+	-	-	-
2	+	-	+	+	-	+	-	+	+	-	-	-
3	-	-	+	-	-	+	-	+	+	-	-	-
4	+	-	+	+	-	+	-	-	+	-	-	-
5	+	-	+	+	-	+	-	-	+	+	-	-
6	+	-	+	+	-	+	+	-	+	-	-	-
7	+	-	+	+	-	-	-	-	+	-	-	-
8	-	-	-	-	-	-	-	+	-	-	-	-
9	-	-	+	-	-	+	-	+	+	-	-	-
Total	67%		78%	56%		67%	11%	44%	89%	11%		
**Positive**												
1	+	-	+	-	-	+	-	+	+	+	+	-
2	+	-	+	-	-	+	-	+	+	+	+	-
3	+	-	+	+	+	+	-	+	+	-	+	-
4	+	-	+	-	-	+	-	+	+	-	-	-
5	+	+	+	+	-	+	-	-	+	-	+	-
6	-	-	-	-	-	-	-	+	+	+	+	-
7	-	-	-	-	-	-	+	+	+	-	-	-
8	-	-	+	-	-	+	-	-	+	-	-	-
9	-	-	+	+	-	+	+	-	+	-	-	-
10	+	-	+	-	-	+	+	-	+	+	-	-
11	-	-	-	-	-	+	-	-	+	+	-	-
12	+	-	+	+	-	+	-	-	+	-	-	-
13	-	-	+	+	-	+	-	-	+	-	-	-
Total	54%	8%	77%	39%	8%	85%	23%	46%	100%	39%	39%	
OR	0.57	18.47	0.94	0.50	18.47	1.94	2.41	1.08	25.82	5.17	129.09	NA
CI	0.32-1.02	1.05-324.5	0.48-1.83	0.28- 0.88	1.05- 324	0.94-4.01	1.10-5.27	0.62-1.89	1.50-444.6	2.45-10.8	7.7-2138.5	NA
P Value	0.06	0.04	0.86	0.01	0.04	0.07	0.02	0.78	0.02	<0.01	<0.01	NA

Oncogenic viruses identified in each VSCC patient as determined by PCR analysis. Most cases tested positive for HPV, EBV, KSHV, and HSV in HIV-1 positive and negative samples. None was positive for JC virus.

### Immunohistochemistry

**Figure 1:**
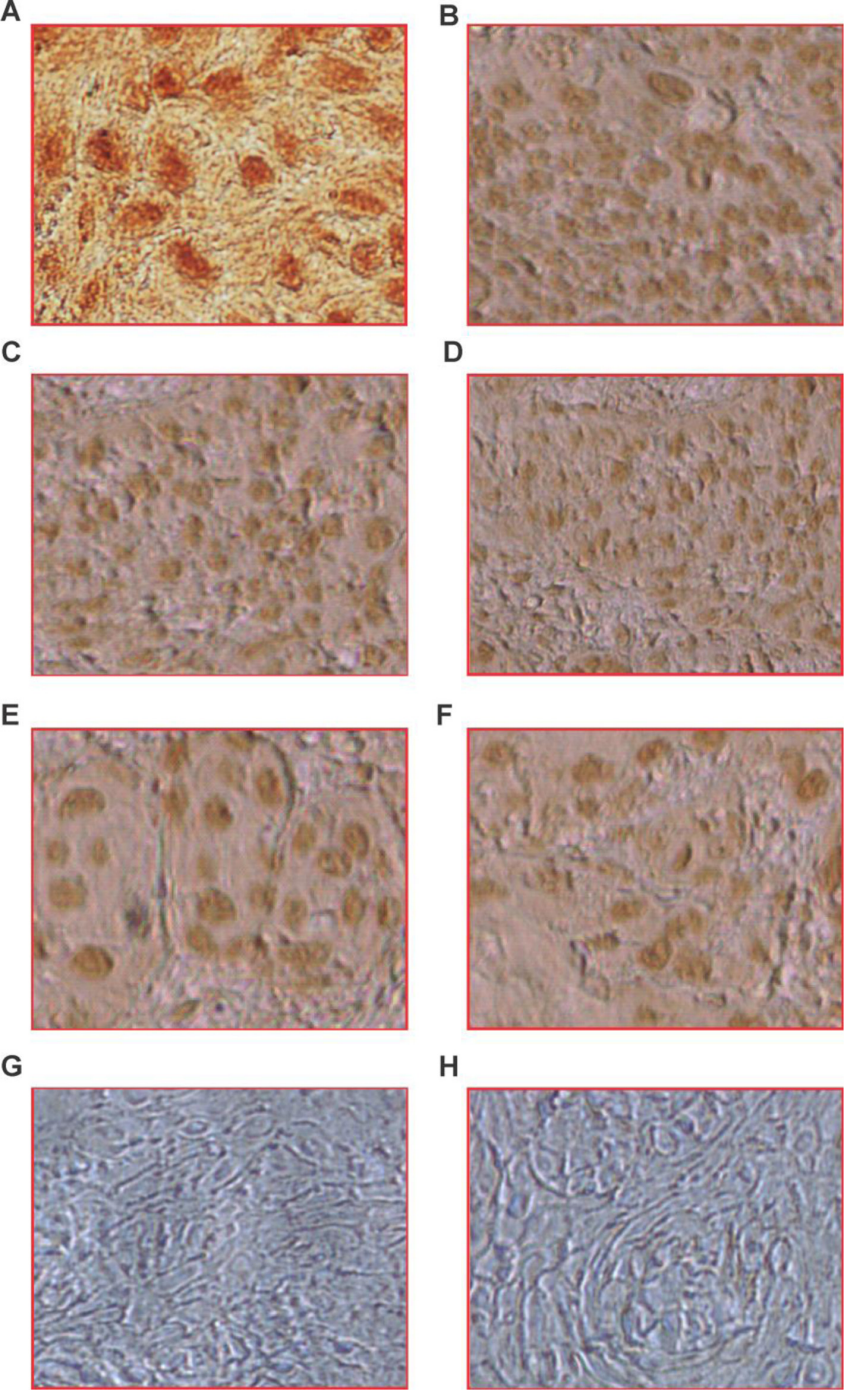
**Representative immunohistochemistry results of selected VSCC tissues.** Panel **A**. shows brown staining positive cells for LMP1 using supernatant from the S12 hybridoma; panel **B**. shows brown staining positive cells using KSHV-LANA antibody; panel **C**. shows brown staining positive cells using HPV-16-E6 antibody; panel **D**. shows brown staining positive cells using HPV-16 E7 antibody; panel **E** shows brown staining using Cyclin D1 antibody; panel **F**. shows brown staining using p21Waf1/Cip1 antibody; panel **G**. shows negative staining without antibody, and panel **H** shows negative sample control. Magnification at 40X.

We performed IHC using a panel of antibodies that included anti LANA in KSHV (Figure 1B), anti-LMP1 in EBV (Figure 1A), and anti HPV-16, E6 (Figure 1C) and E7 (Figure 1D). We also used antibodies against the cell cycle proteins P21 (Figure 1F), Cyclin D1 (Figure 1E), and VEGF. We observed that LANA was expressed in a larger percentage of positive HIV-1 samples (61%) compared to the negative samples (44%) (OR = 1.99, CI = 1.13-3.49, P = 0.01) (Table 3). The ubiquitous EBV, noted earlier in our previous paper [23] was highly expressed in more negative cases (100%) compared to the HIV positive cases (69%) (OR = 0.01, CI = 0.01-0.18, P = 0.001). Surprisingly, HPV-16 E6 was detected in more negative samples (56%) than positive samples (31%) (OR = 0.35, CI = 0.19-0.63, P = 0.004). HPV-16 E7 was similar in both negative and positive samples (77%) (OR = 1.0, CI = 0.51-1.93, P = 1.0). The P21 cell cycle marker was detected in more positive samples (77%) than in negative samples (44%) (OR = 4.26, CI = 2.31-7.84, P < 0.001). Cyclin D1 was expressed in more negative samples (88%) than positive samples (54%) (OR = 6.24, CI = 3.04-12.83, P < 0.001) while VEGF signals were detected in 89% of negative samples and 92% of the positive samples (OR = 1.42, CI = 0.54-3.6, P = 0.47) (Table 3).

**Table 3:** Immunohistochemistry summary of HIV-1 negative and positive vulva cancer samples

**Negative**	**IHC-LANA**	**LMP1**	**Cyc. D1**	**HPV16-E7**	**HPV16 E6**	**p21Waf1/Cip1**	**VEGF**
1	-	+	+	+++	+	+	-
2	-	+	+	-	+	++	+++
3	-	+	-	-	+	-	+
4	+	+	+	+	-	+	++
5	+	+	++	++	-	-	++
6	+	+	++	++	-	-	+++
7	-	+	+	+	+	+	+
8	+	+	+	+	+	-	+
9	-	+	+	+	-	-	+
Total	44%	100%	88%	77%	56%	44%	89%
**Positive**							
1	-	-	+	-	-	-	-
2	+	+	+	+	-	+	+
3	-	-	-	-	-	-	+
4	-	+	+	+	-	+	+
5	-	-	-	+	-	+	++
6	+	+	+	++	+	++	+
7	-	-	-	-	-	-	+
8	+	+	-	+	+	+	++
9	+	+	+	++	+	++	++
10	+	+	-	+	+	+	+
11	+	+	+	+++	-	+++	++
12	+	+	+	+	-	+	++
13	+	+	-	+	-	+	++
Total	61%	69%	54%	77%	31%	77%	92%
OR	1.99	0.01	0.16	1.0	0.35	4.26	1.42
CI	1.13-3.49	0.01-0.18	0.07-0.32	0.51-1.93	0.19-0.63	2.31-7.84	0.54-3.6
P value	0.01	0.001	<0.01	1.00	0.004	<0.001	0.47

Oncogenic viruses identified in each VSCC patient as determined by Immunohistochemistry analysis. Most cases tested positive for KSHV, EBV, Cyclin D1, HPV16-E7, HPV16-E6, p21, and VEGF.

### Multiple HPV types detected in vulvar samples

GP5+/GP6+ and specific HPV primers were used for our PCR amplification. GP5+/GP6+ primer can detect at least 20 HPV types and has been used widely in clinical assays [19,20,23]. In our experiments using these primers we detected multiple HPV types in the VSCC tissues. The results showed that high risk HPV-16, 18, 45, 31, and 33 and low risk HPV-6 and 11 were prevalent in our small sample, a possible representation of the general population prevalence (Table 2).

### We detected high risk HPV in vulvar tissues by PCR and IHC

Utilizing PCR and IHC, high risk HPV-16 (OR = 1.05, 95% CI = 0.54-2.05, P = 0.86); 18 (OR = 0.05, 5% CI = 0.03-9.09, P = 0.046); 45 (OR = 2.3, 95% CI = 1.3-4.03, P = 0.003); 33 (OR = 1.05, 95% CI = 0.54-2.05, P = 0.86); and 31(OR = 0.35, 95% CI = 0.17-0.71, P = 0.003) were detected in the tissues of patients (Table 2). The level of expression varied from patient to patient, however it was observed that HPV-16, 31, and 45 were the most frequently detected (Table 2). HPV-16 E6 and E7 (Figures 1C,D) were detected in tissues by IHC. Of interest was the detection of these viral types in both the negative and positive cases almost to equivalent levels suggesting similar exposure to these agents in HIV positive and negative individuals.

### The ubiquitous agent EBV was detected at similar levels in VSCCs and controls

EBV DNA was detected to almost equal levels in controls and VSCC cases underscoring the ubiquity of the virus in the population by PCR (OR = 0.03, 95% CI = 0.02-0.66, P = 0.02) (Table 2), and by IHC (OR = 91, 95% CI = 5.4-1513, P = 0.001) (Table 3). The EBV viral genetic DNA was detected in the tissues with other herpesviruses such as HPV and KSHV. The similar levels of EBV in controls and cases, and the possible association of EBV in initiation of VSCC in HIV-1 infected cases warrants further investigation.

### KSHV was detected mostly in HIV positive VSCC compared to HIV negative VSCC control samples

KSHV, another oncogenic gammaherpesvirus that has been associated with Kaposi’s sarcoma (KS) was identified in positive cases more than in the negative controls by PCR (OR = 0.193, 95% CI = 0.09-0.41, P < 0.001) (Table 2) and by IHC (OR = 0.502, 95% CI = 0.28-0.88, P = 0.05) (Table 3). Even though the findings are from a small sample, we noted similar results [23] with EBV, KSHV, and HPV types equally represented in the cancerous tissues suggesting further investigation on effects of oncogenic viral interactions in initiation and maintenance of the cancers.

### Expression of cell cycle protein markers p21 and Cyclin D1, as well as VEGF in vulvar samples as detected by IHC

We assayed for the cell cycle regulators p21 (Figure 1F) and Cyclin D1 (Figure 1E), as well as VEGF in the tissue samples using IHC. It was observed that p21 was significantly higher in cases than in controls (OR = 0.23, CI = 0.21-0.43, p < 0.001) while Cyclin D1 was higher in the negative controls (OR = 6.24, CI = 3.04-12.83, P < 0.001). Interestingly, VEGF signals were similar in controls and in VSCC cases (OR = 0.70, CI = 0.27-1.83, P = 0.47), respectively (Table 3).

### The PCR assay did not detect JC virus used as a negative control for the samples

John Cunningham (JC) virus [18], which has been identified mostly in transplant patients, was also tested for its presence in the samples. No JC virus signals were detected in the controls nor the VSCC cases (Table 2).

### CD4 counts and the cell cycle proteins

CD4 has been used as a marker of immunocompetence in HIV infected patients, with higher CD4 counts indicating better immune response in these patients to opportunistic infections and other pathogenic infections. We assessed CD4 counts in relation to Cyclin D1 in the VSCC cases and controls. We observed that in the HIV positive VSCC cases the counts were 437.9 compared to 409.8 for the controls. For p21 signals the CD4 counts were 410 for the HIV positive cases and 457 for the HIV negative controls. For VEGF positive samples CD4 counts were 430.1 for the HIV positive cases and 477 for the HIV negative controls.

### HAART therapy may contribute to the progression of VSCC in HIV positive patients

The CD4 range for HIV positive individuals who were not currently taking HAART average was 392 compared to HIV positive cases taking HAART with an average of 457.5.

There was a significant difference between cases who were currently on HAART and those not taking HAART (OR = 152.1, 95% CI = 9.1-2516.9, P = 0.0005). When comparison was made between HAART therapy and human papilloma virus detection by PCR HPV-18 (OR = 0.0083, 95% CI = 0.0001-0.50, P = 0.02), HPV-33 (OR = 0.0083, 95% CI = 0.0001-0.50, P = 0.02), HPV-31 (OR = 121, 95% CI = 2.02-7259.7, P = 0.02), HPV-6 (OR = 0.0083, 95% CI = 0.0001-0.50, P = 0.02), as well as oncogenic herpesviruses EBV (OR = 121, 95% CI = 2.02-7259.7, P = 0.2), and KSHV (OR = 0.0083, 95% CI = 0.0001-0.50, P = 0.02) were significant while HPV-16 (OR = 16, 95% CI = 0.7215-354.8, P = 0.07), HPV-45 (OR = 2.25, 95% CI = 0.17-28.3, P = 0.53), and HPV-11 (OR = 0.06, 95% CI = 0.0028-1.39, P = 0.08) were borderline significant (Table 2).

### Age may have significant role in vulvar cancer in Batswana women who are HIV positive

It was observed that the HIV positive cases with vulvar cancer were typically younger with a median age of 38 years while the HIV negative cases were generally older, median age 62 years (OR = 0.3757, 95% CI = 0.2122-0.66650, P = 0.0008). This strongly suggests that in vulvar cancer, HIV may be contributing to the initiation of the cancer in younger females through pathways that have not been fully delineated (Table 1).

### ARV therapy and cell cycle protein expression

When we looked at the expression of cell cycle proteins in cases on HAART compared to controls, we observed no significant difference for Cyclin D1 (OR = 2.25, 95% CI = 0.18-28.26, P = 0.53), whereas there were significant differences for p21 (OR = 121, 95% CI = 2.02-7259.7, P = 0.02). For the growth factor VEGF (OR = 121, 95% CI = 2.02 = 7259.7, P = 0.02) also showed a significant difference for women who are on HAART compared to controls (Table 3), though due to the small sample size it may be important to evaluate this question with a larger sample size.

## Discussion

Studies indicate that a majority of keratinizing squamous cell carcinomas usually found in older women are likely to be non-HPV related [24,25]. Yet, due to other factors such as cigarette smoking, multiple sex partners, and other infections, women over 60 years can have HPV-associated vulvar cancers [26]. In their study Madeleine et al. observed high prevalence of HPV-16, and less prevalence of HPV-18 and HPV-6 [26]. The authors also indicated that HSV-2 may contribute to vulvar cancer independently of HPV-16, through another yet to be identified pathway. This may be routed in the stimulation of the immune response as a result of the HSV infection. Our study showed a similar trend, with more detection of HPV-16 than HPV-18 and other HPV-types in both patients younger than 50 years and older who were HIV-1 infected, with the younger HIV positive cases having increased risk of having VSCC than older cases. We observed a high incidence of EBV in both the HIV-1 negative and HIV-1 positive samples. Interestingly, as it would be expected for opportunistic infections, more KSHV was detected in the HIV-1 positive cases than HIV-1 negative cases by both PCR and immunohistochemistry. These findings were not entirely surprising since we observed similar distribution of these viruses in Ocular surface squamous neoplasia (OSSN) tissues from same population in Botswana [23]. The implications of detection of oncogenic viruses from the small sample size calls for more investigation. HPV was detected in positive samples as well as in controls. It is likely that in our cohort contributing factors to VSCC initiation and maintenance may include HIV-I and other infections that elicit inflammatory responses and also cause immunosuppression, besides HPV.

The viral genes of HPV-16, E6 and E7 have been shown to interfere with 2 pathways associated with cell cycle deregulations. E6 has been shown to interact with p53, causing p53 dysfunction and eventually disrupting cell cycle arrest [27]. HPV-16 encoded E7 inactivates pRb leading to overexpression of p16^INK4A^ resulting in hyperproliferation that can be detected by antibodies against Ki-67 nuclear antigen present in all cell cycle stages of proliferating cells except for the G_0_ phase that was expressed to a lower level than HPV-16 E7 in our samples (Figure 1D) [27]. The range in expression could be due to viral load of both HIV-1 and HPV types and associated immunosuppression. The HIV-1 viral load depended on when HAART therapy was initiated in each patient. In our study some of the patients were on HAART, however, because of the sample size it was difficult to decipher the effect of HAART that could be generalized, though significant differences were observed between those on HAART and those not on HAART at the time of data collection. Chaturvedi et al. [28], observed increased incidence, prevalence, and persistence of HPV infections and cofactors such as cigarette smoking among HIV-1 infected patients which led them to hypothesize that low CD4 count during the pre-HAART era masked the association between immunosuppression and the risk of HPV related invasive cancer that increased survival during the HAART era providing adequate time for progression of premalignant lesions to invasive cancers as suggested in our paper [23]. Chaturvedi et al. suggest that poor immune control of premalignant lesions that may facilitate the development of cancer and later progression to invasive cancer is not affected by immunosuppression [28]. HAART has been shown not to alter HPV persistence or rates of progression or regression of premalignant anogenital lesion [29,30]. Palesfky et al. suggest that the increase in anal cancer in the HAART era may reflect the possibility that in the pre-HAART era patients at highest risk of anal cancer would have died from other causes [29,30]. Since the introduction of large scale HAART therapy in Botswana took place in 2002 (WHO, 2006), most of the patients who had these oncogenic neoplasias pre-HAART era died without diagnosis or therapy.

Perturbation of cell cycle proteins was also considered a factor in VSCC, thus we assayed for Cyclin D1 which is in the p53 pathway, p21 which is downstream target of p53, and VEGF whose contribution to vascularity is considered essential for tumor progression [9,13-15,31,32]. Our observation of significant differences in p21, and Cyclin D1, and no difference in VEGF necessitates a more robust and broader study with a larger sample size. However, these results do provide new evidence for a role of oncogenic viruses in development of VSCC.

## Conclusion

Though our sample size is small we do suspect that similar immunological surveillance dysfunction and dysregulation of specific pathways associated with viral oncogenesis may be a factor in vulvar carcinoma pathogenesis. Additional levels of investigation are needed in HIV-1 associated vulvar carcinoma to delineate the dysregulation these viruses will initiate that leads to oncogenesis.

The findings of different oncogenic viruses and expression of cancer associated markers in tissues of women with and without HIV in Botswana with VSCC warrants further investigation with a larger sample size that may lead to identification of molecular moieties for targeted therapeutic and vaccine development. This Botswana cohort was unique in exposure to different environmental and infectious agents that may have additional epigenetic effects on the outcome of VSCC [33].

## Competing interests

The authors declare that they have no competing interests.

## Authors’ contributions

KOS designed the study, conducted experiments, interpreted data and drafted the manuscript and supervised the study, HCJ interpreted data and drafted the manuscript, MK identified VSCC tissues, CK identified VSCC tissues, and ESR designed the study, interpreted data, edited the manuscript, and supervised the study. All authors have read and approved the final manuscript.
